# Spinal Injuries in the 2012 Twin Earthquakes, Northwest Iran

**DOI:** 10.1371/currents.dis.39b14d88c93fe04ef1a2ce180b24f8d1

**Published:** 2013-03-27

**Authors:** Kamyar Ghabili, Samad E J Golzari, Firooz Salehpour, Taghi Imani, Amir Mohammad Bazzazi, Alireza Ghaffari, Hadi Mohammad Khanli, Parastou Tizro, Shabnam Taghizade, Seyed Kazem Shakouri

**Affiliations:** Physical Medicine and Rehabilitation Research Center, Tabriz University of Medical Sciences, Tabriz, Iran; Cardiovascular Research Center, Tabriz University of Medical Sciences, Tabriz, Iran; Students’ Research Committee, Tabriz University of Medical Sciences, Tabriz, Iran; Department of Neurosurgery, Tabriz University of Medical Sciences, Tabriz, Iran; Department of Neurosurgery, Tabriz University of Medical Sciences, Tabriz, Iran; Department of Neurosurgery, Urmia University of Medical Sciences, Urmia, Iran; Medical Philosophy and History Research Center, Tabriz University of Medical Sciences, Tabriz, Iran; Faculty of Medicine, Tabriz University of Medical Sciences, Tabriz, Iran; Faculty of Medicine, Tabriz University of Medical Sciences, Tabriz, Iran; Faculty of Medicine, Tabriz University of Medical Sciences, Tabriz, Iran; Physical Medicine and Rehabilitation Research Center, Tabriz University of Medical Sciences, Tabriz, Iran

## Abstract

On 11 August 2012, twin earthquakes measured 6.3 and 6.4 on the Richter scale hit three towns (Ahar, Varzaqan, and Heris) in East Azerbaijan Province, Iran resulting in tragic loss of three hundred lives and leaving thousands of injured. The aim of the present study was to report the spinal injuries during recent earthquake in northwest Iran, its consequences and management. Of the 923 hospitalized patients, 26 (2.8%) had neurosurgical complications. The imaging and clinical data of the patients were retrospectively studied regarding the anatomical location of the injury, the severity of spinal injury and associated neurological deficit. To further analyze the findings, Magerl (AO) and Frankel’s classifications were used. The injuries without any fracture were considered as minor spinal injuries. The mean age of the patients was 44.54±22.52 (range: 5-88) years. We detected a total of 38 vertebral injuries including 24 major (63.15%) and 14 minor injuries (36.85%). The most common injuries were observed in the lumbar spine (19 injuries, 50%). The 24 major injuries chiefly included Magerl type A (14 injuries, 58.3%). According to the Frankel’s classification, majority of the patients (88.46%) had no neurological deficit. In this study, three patients had nerve injuries. In conclusion, the number and proportion of spinal fractures patients in the recent twin earthquakes, northwest Iran was limited and caused less nerve injuries compared to the previous similar disasters. This might be due to the milder earthquake consequences since the incident happened in the middle of the day when men were working their fields. Potential complications in patients traumatized in earthquake incidents should be monitored for and early assessment of the neurological function is required to prioritize care for the victims.

## Introduction

Earthquakes are one of the most catastrophic natural disasters leading to dramatic casualties. Adequate and appropriate management of the injured in earthquakes is of immense concern to rescuers and medical-care providers in order to reduce mortality and morbidity.^1^ ^2^ Proper allocation of medical resources and enhanced implementation of medical assistance would be guaranteed by better understanding and evaluation of the injury characteristic in earthquakes.^3^

On 11 August 2012, twin earthquakes measured 6.3 and 6.4 on the Richter scale hit three towns (Ahar, Varzaqan, and Heris) in East Azerbaijan Province, Iran resulting in tragic loss of three hundred lives and leaving thousands of injured.^4^ ^5^ ^6^ The victims trapped under the rubble were rescued following the event by the rescue teams and the survivors were housed in the provided emergency shelters. A total of 961 severely injured were transferred to the nearest referral hospitals in Tabriz, the capital of East Azerbaijan Province, of which 38 died.^7^ Overall, 2.8% of all hospitalized patients had spinal injuries which were diagnosed and treated appropriately. The aim of the present study was to report the spinal injuries during recent earthquake in northwest Iran, its consequences and management.

## Methods

Of the 923 hospitalized patients, 26 had neurosurgical complications. The imaging and clinical data of the patients were retrospectively studied regarding the anatomical location of the injury, the severity of spinal injury and associated neurological deficit. To further analyze the findings, Magerl (AO) and Frankel’s classifications were used.^8^ ^9^ According to the Magerl (AO) classification, type A (compression injuries of the anterior column) is subdivided into A1 (impaction fractures), A2 (split fractures), and A3 (burst fractures); type B (distraction injuries of the anterior and posterior columns with transverse disruption) is subdivided into B1 (posterior disruptions that are predominantly ligamentous), B2 (posterior disruptions that are predominantly osseous), and B3 (anterior disruptions through the disk); and type C (anterior and posterior element injuries with superimposed rotation resulting from axial torque) is subdivided into C1 (type A injuries with rotation), C2 (type B injuries with rotation), and C3 (rotational-shear injuries).^8^ Neurologic function according to Frankel’s classification was evaluated as grade A (complete); grade B (sensory only); grade C (useless motor power without motor function); and grade D (useful motor power without functional movement).^9^ The injuries without any fracture were considered as minor spinal injuries. The Tabriz University of Medical Sciences Institutional Review Board approved the project and investigators followed the principles of the Declaration of Helsinki. Written informed consent was obtained from patients or their guardians.

## Results

Based on the clinical records, 26 patients (12 males and 14 females) had neurosurgical injuries during the recent earthquake in northwest Iran. The mean age of the patients was 44.54±22.52 (range: 5-88) years. We detected a total of 38 vertebral injuries including 24 major (63.15%) and 14 minor injuries (36.85%). The most common injuries were observed in the lumbar spine (19 injuries, 50%) followed by thoracic (8 injuries, 21.06%), cervical (6 injuries, 15.79%), and sacral (5 injuries, 13.15%) spine. The 24 major injuries chiefly included Magerl type A (14 injuries, 58.3%) (Figure 1). No combined cases of Magerl type A and B or C were observed. Odontoid and occipital condyle fractures were observed in 2 and 1 cases, respectively. According to the Frankel’s classification, majority of the patients (88.46%) had no neurological deficit (grade E). Moreover, 7.69% and 3.84% of the patients were classified as grade D (useful motor power without functional movement) and C (useless motor power without motor function), respectively.

**Classification of the 24 major spinal injuries according to the Magerl (AO) types:**
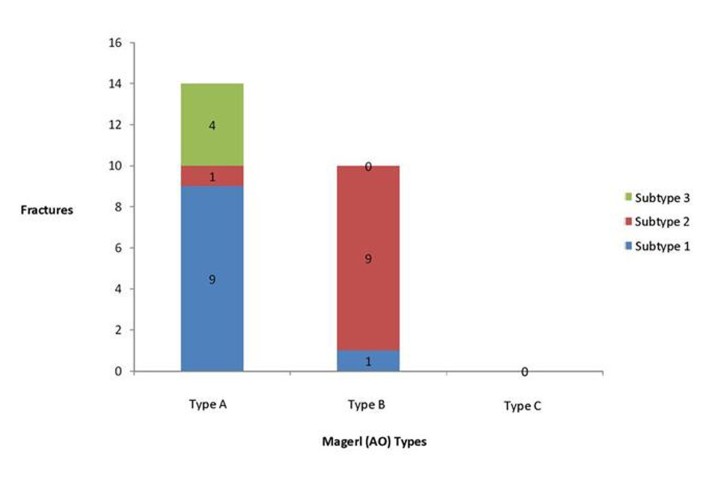

In this study, three patients had nerve injuries; patient 1 (35 years old, female, fracture of four lumbar vertebrae at transverse process), patient 2 (77 years old, male, fracture of first lumbar and twelfth thoracic vertebrae), and patient 3 (24 years old, female, sacral fracture). Subsequently nine patients (34.61%) were managed with thoracolumbar support belt, 5 patients (19.23%) with lumbosacral support belt, 5 patients (19.23%) with cervical collar, 4 patients (15.38%) with the Cotrel-Dubousset (CD) instrumentation, 2 patients (7.69%) with conservative approaches, and 1 patient (3.84%) with halo cast.

## Discussion

The present study revealed that the most common region for spinal injuries was the lumbar spine. This finding is similar to that of the studies on the neurosurgical complications during the previous earthquakes.^3^ ^10^ ^11^ ^12^ In studies performed after the Sichuan, China earthquake in 2008, 40-55% of the patients with spinal injuries were reported to suffer from lumbar involvement.^10^ ^11^ ^12^ Interestingly, in a similar report after Yushu, China earthquake in 2010, Li and colleagues detected lumbar fracture in almost 70% of the patients with spinal fractures.^3^ Furthermore, our study showed that major spinal injuries mainly included Magerl type A (58.3%). This finding is consistent with that of the similar study by Dong et al. indicating that 61.5% of the major injuries consisted of Magerl type A injuries.^11^

In the present study, neurological deficit occurred in 11.5% of the patients which is less than the corresponding figures (20-30%) in the Sichuan earthquake in 2008.^10^ ^11^ ^12^ In addition, none of our cases with cervical injuries were associated with neurological deficit which is sharply in contrast to the related literature.^10^ In the present study, three patients with nerve injuries had fractures at different vertebrae including thoracic, lumbar, and sacrum. However, we could not reach any conclusion on the association between the nerve injury and the level of spinal fracture due to the limited number of patients with nerve injuries following the spinal fractures. Li et al. found that all their patients with nerve injuries had lumbar spine fracture.^3^ In contrast, thoracic injury contributed to the majority of the cases with neurologic injury in the study by Chen and colleagues following the 2008 Sichuan, China earthquake.^10^

In conclusion, the number and proportion of spinal fractures patients in the recent twin earthquakes, northwest Iran was limited and caused less nerve injuries compared to the previous similar disasters. This might be due to the milder earthquake consequences since the incident happened in the middle of the day when men were working their fields. Potential complications in patients traumatized in earthquake incidents should be monitored for and early assessment of the neurological function is required to prioritize care for the victims.

## Competing Interests

The authors have declared that no competing interests exist.

## Correspondence

Corresponding Author: Dr. Samad EJ Golzari. Email: dr.golzari@hotmail.com
